# Brief Report: Parent Perspectives on Sensory-Based Interventions for Children with Autism Spectrum Disorder

**DOI:** 10.1007/s10803-020-04644-8

**Published:** 2020-08-27

**Authors:** Moira Peña, Yvonne Ng, Jacquie Ripat, Evdokia Anagnostou

**Affiliations:** 1 Holland Bloorview Kids Rehabilitation Hospital, 150 Kilgour Road, Toronto, ON Canada; 2 Department of Occupational Science & Occupational Therapy, University of Toronto, Toronto, ON Canada; 3 Department of Occupational Therapy, College of Rehabilitation Sciences, University of Manitoba, R106-771 McDermot Avenue, Winnipeg, MB Canada; 4 Department of Pediatrics, University of Toronto, Toronto, ON Canada

**Keywords:** ASD, Sensory, Sensory Processing, Sensory-Based interventions, Parent perspectives, Challenging behaviours, Autism

## Abstract

Sensory-Based Interventions (SBIs) are often recommended to enable function/participation in children with ASD. Still, there are limited studies to evaluate their effectiveness. Acceptability studies are an important step towards establishing effective interventions. We examined parents' perceptions of the uptake and acceptability of such interventions. An online survey was sent to 399 families; response rate was 39%. The most frequently therapist-recommended interventions were trampoline (54.6%), massage (47.8%), and oral-motor tools (43.8%). Highest use was reported for massage (96.3%), trampoline (89.2%) and joint compressions and brushing (89.2%). The majority of parents viewed these interventions to be very important /important, (74.8%) but barriers to their use were identified. High acceptability of SBIs was reported by parents of children with ASD.

## Introduction

Atypical sensory processing skills in children with autism spectrum disorder (ASD) have been well established in the literature with up to 96% of children diagnosed with ASD demonstrating sensory processing deficits (Watling et al. 2018). As such, hyper and hypo responsivity to sensory input are now a diagnostic criterion of Autism Spectrum Disorder (*Diagnostic and Statistical Manual of Mental Disorders- Fifth Edition (DSM-5)* (American Psychiatric Association 2013). Although sensory processing differences and their effects on functional performance and participation in daily activities are now widely recognized, Sensory-Based Interventions (SBIs) remain understudied and have yet to be established as effective interventions to target challenging behaviours in children with ASD.

The concept of sensory processing dysfunction in children is based on Dr. A. Jean Ayres’ research and theory development in the mid 1960′s (Watling and Hauer 2015). She noted that some children’s learning challenges, motor delays and atypical behaviours originated from sensory-based impairments and began to develop what is presently known to occupational therapists as the Sensory Integration (OT-SI) approach or theory (Ayres 1979; Reynolds et al. 2017). Occupational therapists use OT-SI theory to make hypotheses about the link between atypical behaviours and neurological processes which are then used clinically to help explain behavior and plan interventions (Miller et al. 2007).

Sensory-Based Interventions (SBIs) are recommended by clinicians who practice occupational therapy using an OT-SI frame of reference (Miller et al. 2007), but do not adhere to all of the core principles of Ayres Sensory Integration® (ASI). While ASI® is a structured treatment process with high fidelity measures that is individualized, child-led and clinic-based (Case-Smith et al. 2015; Schoen et al. 2019), SBIs are therapist or adult-led, take place in the child’s home, school or community environments and can be passively applied in a similar manner across participants (Watling and Hauer 2015). In addition, SBIs may provide either a single sensory or multisensory stimulation and may also include environmental modifications, for example, the relatively common practice of recommending noise cancelling headphones to mitigate noxious auditory input experienced by children with ASD and auditory hyper-responsivity (Yunus et al. 2015; Reynolds et al. 2017). The presumed mechanism of SBI includes a short-term alteration of a person’s physiological state of arousal by decreasing the sympathetic nervous system's activity and increasing parasympathetic responses with the goal of positively influencing attention, behaviour or function (Case-Smith et al. 2015; Watling et al. 2011; Watling and Hauer 2015). Such manipulations are thought to increase children's ability to participate in meaningful activities at home, school and community activities (Reynolds et al. 2017).

Despite the common use of SBIs to address behavioural challenges in children with ASD (Case-Smith and Arbesman 2008; Case Smith et al. 2015), research studies on the effectiveness of SBIs continue to be limited and are mostly open label in nature. There is however preliminary evidence to suggest that some SBIs may be effective in reducing challenging behaviours in children with ASD, particularly when using tactile modalities such as massage (Yunus et al. 2015). Other SBIs that are recommended by occupational therapists but are yet to be supported by the literature (Case-Smith et al. 2015; Weitlauf et al. 2017) include the use of weighted wearables such as a weighted vest (Fertel-Daly et al. 2001; Hodgetts et al. 2011), backpack, blanket or toy, ankle and wrist weights, the use of compression garments such as a compression/pressure vests as well as other tools that may be used to provide sensory input either in singular form or in combination such as the provision of joint compressions and brushing (Fazlioglu and Baran 2008; Weeks et al. 2012), the use of the trampoline as well as oral-motor tools. Robust research studies to evaluate the effectiveness of such interventions are needed; however well-articulated research frameworks addressing pathways to generating efficacy data highlight the value of assessing acceptability as an early research activity that can guide future efficacy studies (Craig et al. 2008; Smith et al. 2007). Given that parents are most likely to be administering these interventions at home or in the community, parents' perceptions of the value, uptake, and acceptability are critical.

## Methods

### Development of the Questionnaire

An online questionnaire was developed to capture demographic information, identification of therapist-recommended SBIs, acceptability of these interventions and barriers to their implementation. The SBIs included in the questionnaire were selected based on the results from a completed literature review by the researchers as well as their clinical experience. The questionnaire was hosted on the secure servers of the Information Systems at Holland Bloorview Kids Rehabilitation Hospital and built using Research Electronic Data Capture (REDcap).

### Data Analysis

Descriptive statistics were used to depict the frequency of use and percentage of parents utilizing the interventions. Open-ended responses were downloaded verbatim into a Word document with the goal of potentially including some of these statements to further clarify the parents’ views if needed, as a full qualitative thematic analysis on the open-ended comments was not within the scope of this study.

### Participants

A convenience sampling method was used to recruit participants for the study. The questionnaire was sent to parents of children with ASD who were registered in the Province of Ontario Neurodevelopmental Disorders (POND) network. POND participants are between 1 and 21 years old, who have been diagnosed with a neurodevelopmental disorder (ASD, attention deficit hyperactivity disorder, obsessive–compulsive disorder, and intellectual disability), or they may be typically developing volunteers. Only the parents of children with a diagnosis of ASD and who were registered at the Holland Bloorview Kids Rehabilitation Hospital's POND site were included in this study.

All participants provided informed consent according to the declaration of Helsinki and institutional guidelines. The study was approved by the Ethic Boards of the University of Manitoba and Holland Bloorview Kids Rehabilitation Hospital.

## Results

### Demographic Information

Three hundred and ninety-nine families were contacted and 152 parents consented to participate in the study yielding an overall response rate of 39%. The majority of the parents (82.6%; n = 123 of 152 responses) noted that their children engaged in a wide range of challenging behaviours including agitation, repetitive, sensory seeking, sensory avoidant, self-injurious, aggressive and poor self-regulatory behaviours which interfered with their ability to participate in daily tasks such as engaging in personal care, school tasks and/or community activities.

### Identification of Sensory-Based Interventions Parents used with their Children

**Fig. 1:**
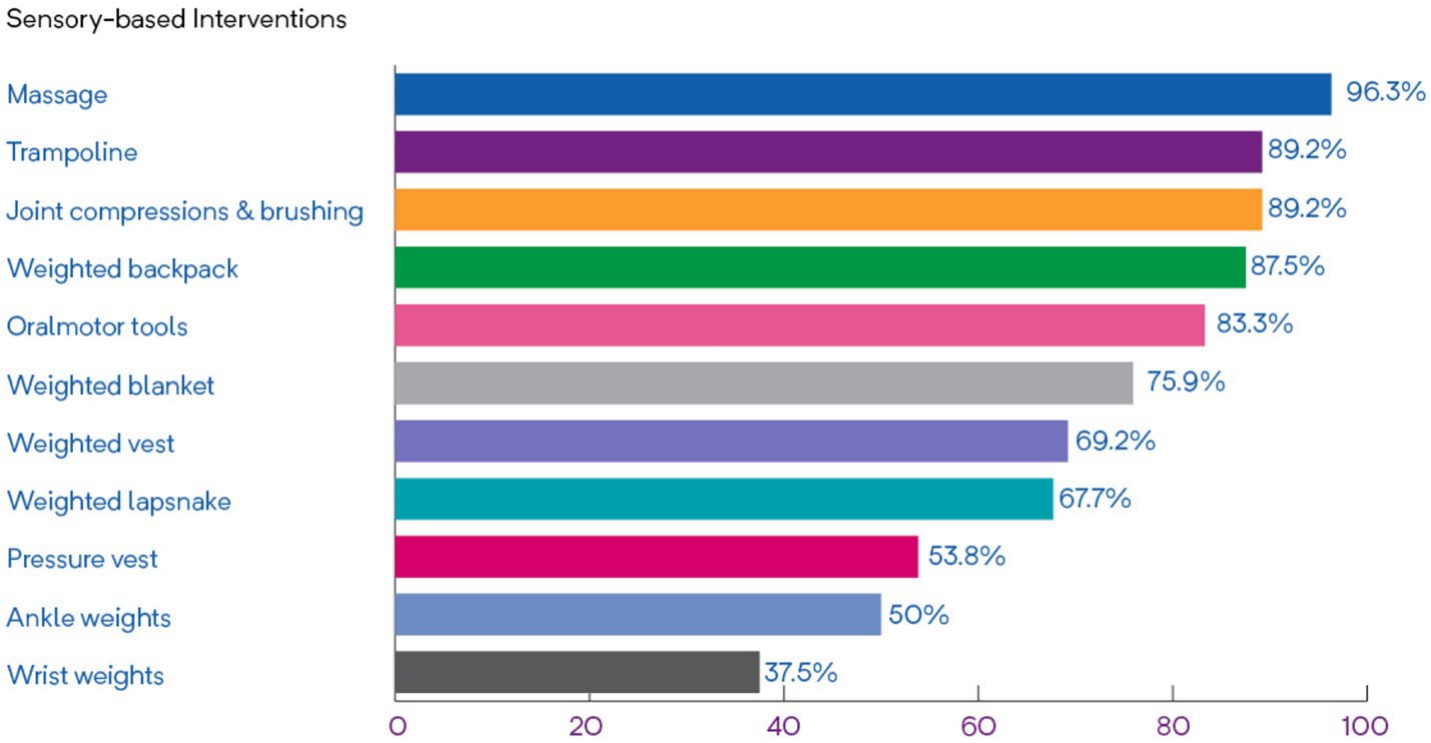
Percentage of recommended sensory-based interventions that are implemented by parents of children with ASD

Parents identified a variety of SBIs that had been recommended to them to address challenging behaviours as well as how frequently these SBIs were being used (Table 1). The use of the trampoline was the most frequently therapist- recommended SBI followed by massage and oral-motor tools. Compression/pressure vests, ankle weights, and wrist weights were the least frequently recommended. When reporting on how frequently the SBIs were being implemented, parents were more likely to use the following: massage, trampoline, and joint compressions & brushing, followed by weighted backpack and oral-motor tools (Fig. 1). Parents were least likely to use wrist weights, ankle weights, and the compression/pressure vest. Of interest, the most consistently used SBIs (i.e., used once a day or more), were weighted backpack (50%), oral motor tools (41.7%), and weighted lap-snake (35.5%) Wrist weights, ankle weights, and compression/pressure vests were reported to be the least frequently used sensory-based interventions.

**Table 1:** Identification of sensory-based interventions (SBI) recommended to parents and how frequently they were used

Interventions	Recommended to parents	Frequency of use of recommended interventions			
		Once a day or more	Twice a week or more	Once a week or less	Never used
Trampoline (n = 116)	65 (56%)^a^	19 (29.7%)^a^	16 (25%)^a^	23 (35.9%)^a^	6 (9.4%)^a^
Massage (n = 113)	54 (47.8%)	14 (25.9%)	12 (22.2%)	26 (48.1%)	2 (3.7%)
Oral-motor tools (n = 112)	49 (43.8%)^b^	20 (41.7%)^b^	8 (16.7%)^b^	12 (25%)^b^	8 (16.7%)^b^
Weighted vest (n = 106)	39 (36.8%)	9 (23.1%)	7 (17.9.9%)	11 (28.2%)	12 (30.8%)
Joint compressions and brushing (n = 111)	37 (33.3%)	11 (29.7%)	6 (16.2%)	16 (43.2%)	4 (10.8%)
Weighted lap-snake and weighted toy (n = 105)	31 (29.5%)	11 (35.5%)	3 (9.7%)	7 (22.6%)	10 (32.3%)
Weighted blanket (n = 106)	29 (27.4%)	9 (31%)	2 (6.9%)	11 (37.9%)	7 (24.1%)
Weighted backpack (n = 103)	16 (15.5%)	8 (50%)	1 (6.3%)	5 (31.3%)	2 (12.5%)
Pressure vest (n = 102)	13 (12.7%)	4 (30.8%)	1 (7.7%)	2 (15.4%)	6 (46.2%)
Wrist weights (n = 102)	8 (7.8%)	0 (0%)	1 (12.5%)	2 (25%)	5 (62.5%)
Ankle weights (n = 101)	6 (5.9%)	1 (16.7%)	0 (0%)	2 (33.3%)	3 (50%)
Other (n = 42)					

*n* number of parents who answered the survey question about whether the specific intervention was recommended to them

^a^Of the 65 respondents only 64 answered the question regarding frequency of use of sensory-based interventions; therefore, the frequency of use percentages were calculated based on the 64 responses

^b^Of the 49 respondents only 48 answered the question regarding frequency of use of sensory-based interventions; therefore, the frequency of use percentages were calculated based on the 48 responses

Some parents (n = 42) reported that they used other SBIs not identified on the questionnaire while others commented on specific characteristics of these SBIs that were collected under the ‘other’ interventions category within the survey. In an exploratory fashion, researchers grouped these open ended responses into three general themes which are worth highlighting, as some parents only answered the open ended questions. The first theme (n = 13) pertained to parents providing sensory input to their children without the use of therapeutic equipment*.* Some of the parents described how they provided firm tactile input to their children through the use of objects that were readily available in their home. For example, parents reported using pillows to make “pillow sandwiches with [child] in the middle”, blankets to make “hot dog rolls”, "tight-fitting clothing", and “chewy foods like bagels and gum” to provide firm tactile input through the jaw area. The second theme pertained to parents providing sensory input through the use of their own body (n = 6). For example, some parents described providing hugs, as one explained “my child asked for input on his body a lot”, and others reported on the use of their hands to squeeze various body parts such as their children's head or abdomen. The third theme referred to parents including heavy work activities into their children's daily routines (n = 4). This was exemplified by statements from parents such as those who reported using a “heavy-work [activities] to prevent issues especially issues with focus and stimming” and “doing hard physical work…such as climbing up the playground slide on her hands and feet – this provided fantastic pressure for her shoulders and hips (which helped her to) pay attention during morning circle… (resulting in) the school adding heavy work activities to the school day”. In addition, a few parents (n = 4) reported they used tactile input combined with movement in a rhythmic manner, such as swinging the child in a sling, bouncing them on a ball, or engaging them in horseback riding.

### Acceptability of Sensory-Based Interventions

**Fig. 2:**
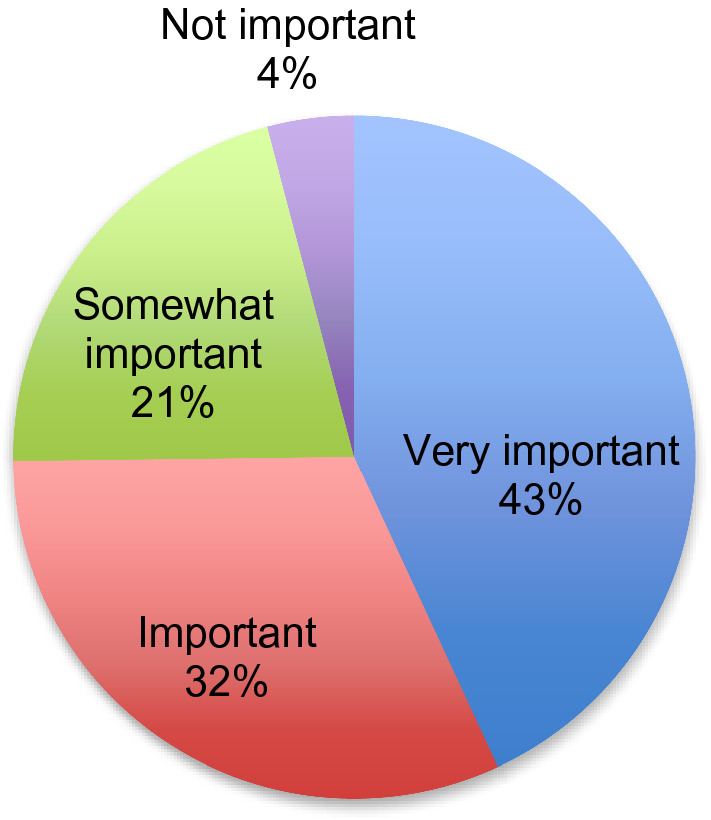
Parents’ perspectives on the importance of sensory-based interventions

From the 123 parents who answered the question relating to the importance of SBI interventions in addressing challenging behaviours, approximately three quarters of respondents stated that they viewed their use to be important or very important as per Fig. 2.

When asked about the helpfulness of the recommended SBIs, more than half of the parents (53.3%) reported that SBIs were helpful in addressing challenging behaviours while 20% of the parents did not find them to be helpful. The remainder (26.7%) reported that SBIs either were sometimes helpful, they were unsure of their effectiveness, or they never had an opportunity to use them.

### Barriers Parents Faced When Trying to Implement Sensory-Based Interventions

**Fig. 3:**
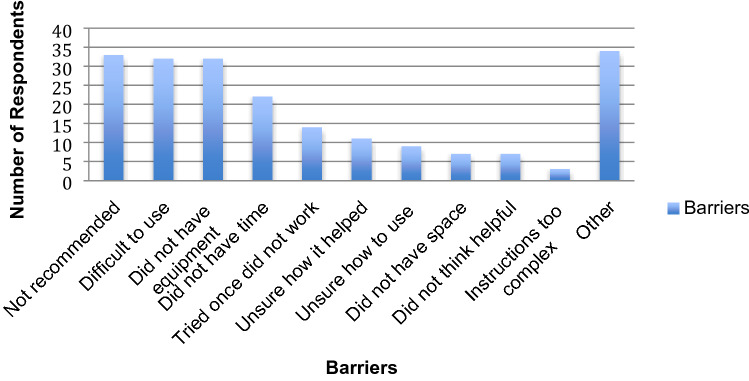
Parents’ perspectives on barriers to using sensory-based interventions. Respondents were asked to choose up to 3 responses

There were many barriers identified by the parents that prevented them from using SBIs. As shown in Fig. 3, parents reported that they found them difficult to use at home and in the community, or they did not have access to the required equipment. In addition, parents reported lack of recommendation as a barrier to accessing such interventions. However, as this study did not evaluate the sensory processing needs of the participants, it is not clear whether such interventions should have been recommended. Parents also identified additional barriers in the ‘other’ open ended question category that were subsequently grouped into two themes: the high cost of the therapeutic equipment and the child’s decreased motivation to engage with the intervention.

In identifying strategies that would make SBIs easier to use, parents emphasized ongoing support from the occupational therapist to be the most helpful followed closely by having access to therapeutic equipment. Some parents identified additional strategies to help increase uptake of these interventions such as: the need for more education and training on the use of SBIs, the need for increased access to affordable equipment, and equipment that is less clinical looking.

## Discussion

SBIs are amongst the most commonly requested services by parents of children with ASD with at least 60% of children receiving SBIs, often as one component of a comprehensive intervention program (Green et al. 2006). SBIs are typically recommended by occupational therapists to increase children's performance and participation in meaningful activities of daily life, such as self-care, school and leisure tasks (Baranek 2002; Reynolds et al. 2017). This is the first known study to explore parents' perceptions of SBIs which is a necessary step towards developing robust efficacy studies in this area (Craig et al. 2008; Smith et al. 2007). The results suggest that parents generally accept and value these types of interventions. A majority of the parents viewed SBIs to be important or very important and indicated that they were helpful in addressing challenging behaviours. However, the parents’ uptake of SBIs varied due to the perceived high cost of equipment, the look of the equipment and being unsure as to when and how to ideally implement these interventions, highlighting gaps for future research. Massage was the most often used SBI by parents to help manage challenging behaviours in their children with ASD, consistent with a growing body of evidence identifying massage as a potentially promising SBI for behavior management in children with ASD (Yunus et al. 2015). The current study also identified some SBIs being used by parents to manage challenging behaviours that have not yet been examined in controlled studies such as the use of the trampoline, oral motor tools, weighted backpack, weighted lap-snake, wrist and ankle weights as well as compression vests. This further emphasizes a disconnect between clinical practice and research, where tools that therapists/parents may find useful have not been systematically evaluated. In addition, this research demonstrates a wide range of frequency of usage of SBIs highlighting the need for future research to focus on identifying protocols and best practice guidelines with regard to the most optimal dosage to ensure the most effective use of these interventions.

Parents in this study indicated that they were more likely to use equipment or objects that were less clinical looking and already found within their households. Occupational therapists are therefore encouraged to carefully consider the types of therapeutic equipment they are recommending. Less reliance on costly sensory equipment and more emphasis on using items readily available within the client’s home, school and community environments where possible may result in increased use of SBIs.

There are several limitations of this study. We include the use of a convenience sample that limits generalization, the use of survey methodology that does not allow for the evaluation of the participants’ sensory processing needs, the survey’s inclusion of only a limited number of SBIs, and the possibility that parents who chose to participate in this study may have had a vested interest in research that is not reflective of the larger population of parents of children with ASD.

## Conclusion

In summary, parents generally accepted and valued SBIs to target challenging behaviours in their children diagnosed with ASD, highlighting SBIs as a set of interventions with high acceptability that warrant further efficacy studies. This research provides clinicians with valuable information as it identifies parental preferences and uptake of a number of SBIs. It also underscores research gaps as several of these SBIs are already employed but understudied.
